# Therapeutic biologics interference in serological and pre-transfusion testing

**DOI:** 10.3389/fimmu.2026.1888597

**Published:** 2026-08-11

**Authors:** Philipp Trummer, Cora P. Habicht, Erwin A. Scharberg, Clemens Schneeweiß

**Affiliations:** 1 imusyn GmbH & Co. KG, Hannover, Germany; 2 SYNLAB MVZ Leinfelden GmbH, Leinfelden-Echterdingen, Germany

**Keywords:** anti-CD20 antibodies, anti-CD38 antibodies, anti-CD47 antibodies, histocompatibility testing, pre-transfusion testing, therapeutic antibody interference, therapeutic biologics interference

## Abstract

The advent of therapeutic antibodies has revolutionized the treatment landscape for various diseases, particularly in oncology and immunology. These highly specific biologic agents bind cell surface proteins, offering targeted therapeutic strategies with potentially fewer side effects compared to traditional treatments. However, the increasing use of these antibodies has introduced new challenges in transfusion and transplantation diagnostics. This review article explores how therapeutic biologics interfere with serological and pre-transfusion testing, focusing on biologics that target CD20, CD38, and CD47, discusses current remedies and solutions to mitigate these interferences, and considers possible interferences from new drugs and drug targets.

## 1 Introduction

### 1.1 The rising tide of therapeutic biologics

Therapeutic antibodies and other biologics are designed to target specific proteins, often of immunological or oncogenic relevance. The success of the first therapeutic antibodies has driven a substantial expansion in research and development, resulting in a rapidly growing arsenal of therapeutic options and an increasing number of patients receiving these drugs. As the use of therapeutic biologics continues to rise, their impact on laboratory medicine has become increasingly apparent. In particular, some therapeutic biologics interfere with diagnostic assays, creating significant challenges in the daily practice of immunohematology and immunogenetic laboratories.

Earlier literature commonly referred to this phenomenon as Therapeutic Antibody Interference. However, not all therapeutic molecules interfering with diagnostics, as discussed in this review, are antibodies. Some are fusion proteins consisting of binding domains linked to the Fc fragment of an antibody, while others lack an Fc domain altogether but nevertheless interfere with diagnostic assays. Therefore, we propose the broader and more accurate term Therapeutic Biologics Interference (TBI).

### 1.2 Anti-CD20: the pioneer

The anti-CD20 antibody rituximab was the third monoclonal therapeutic antibody approved by the Food and Drug Administration (FDA) in 1997 and the first monoclonal antibody developed for treating B cell non-Hodgkin lymphoma (NHL) (1, 2). It soon became well known for its efficacy in treating chronic lymphocytic leukemia (CLL) (3) and a variety of autoimmune diseases (4, 5) and has since revolutionized the treatment of B cell malignancies. Additionally, rituximab is used as off-label medication to improve outcomes in ABO-incompatible kidney transplantation (6). The success of rituximab has paved the way for the development of newer anti-CD20 antibodies, such as ofatumumab or obinutuzumab, with enhanced efficacy (7), optimized modes of action (8), or reduced immunogenicity (9).

In general, anti-CD20 antibodies act through several mechanisms, including complement-dependent-cytotoxicity (CDC), antibody-dependent cellular cytotoxicity (ADCC), and direct apoptosis (10–12). Depending on their ability to reorganize CD20 into lipid rafts, anti-CD20 antibodies can be classified into two categories, type I and type II. By promoting CD20 clustering, type I antibodies such as rituximab and ofatumumab can efficiently recruit proteins of the classical complement cascade, hence exerting potent CDC (13). Type II antibodies, such as obinutuzumab, are lacking the ability to induce CD20 clustering and show decreased CDC activity. However, they are more effective in inducing direct cell death compared to type I antibodies (14).

Therapies with anti-CD20 antibodies have long been established. Nevertheless, more and more anti-CD20 drugs are being developed and brought to market (see **Table 1**). Often, these anti-CD20 drugs are bispecific CD20×CD3 antibodies, also called Bispecific T-cell Engager (BiTE), that recruit T cells to the target. Prominent examples for bispecific antibodies are mosunetuzumab (15) and epcoritamab (16), both of which have been approved by the FDA and the European Medicines Agency (EMA).

**Table 1:** Anti-CD20 drugs approved and in clinical development.

Anti-CD20 drug	Description	Manufacturer	Most advanced clinical status	Indication	Interference
Rituximab (origin clone murine AB 2B8) Rituxan, MabThera	Type I Chimeric monoclonal anti-CD20 antibody; IgG1	Genentech (Roche) Biogen	Approved	B-cell Non-Hodgkin Lymhpoma (B-cell NHL) Chronic Lymphocytic Leukaemia (CLL) Rheumatoid Arthritis (RA) And others	FCXM CDCXM
Ofatumumab (Clone 2F2) Kesimpta, Arzerra	Type I Human monoclonal anti-CD20 antibody; IgG1	Novartis	Approved	Relapsing Multiple Sclerosis (RMS) – Kesimpta Chronic Lymphocytic Leukaemia (CLL) – Arzerra	FCXM CDCXM
Ublituximab (TGTX-1101/LFB-R603) Briumvi	Type I Glycoengineered chimeric monoclonal anti-CD20 antibody; IgG1	LFB Group TGT Therapeutics (licensed)	Approved	Relapsing Multiple Sclerosis (RMS) Chronic Lymphocytic Leukaemia (CLL) – Phase III	FCXM CDCXM
Obinutuzumab (GA101) Gazyva	Type II Humanized, glycoengineered monoclonal anti-CD20 antibody; IgG1	Genentech (Roche)	Approved	Chronic Lymphocytic Leukaemia (CLL) Follicular Lymphoma (FL) Lupus Nephritis (LN)	FCXM Was shown not to affect CDCXM at low concentration (17)
Ocrelizumab (PR-070769) Ocrevus	Type I Humanized monoclonal anti-CD20 antibody; IgG1	Genentech (Roche)	Approved	Primary Progressive Multiple Sclerosis (PPMS) Relapsing Multiple Sclerosis (RMS)	Unknown, but likely CDCXM
Mosunetuzumab (RG7828) Lunsumio	Humanized monoclonal bispecific anti-CD20/anti-CD3 antibody	Genentech (Roche)	Approved	Relapsed or Refractory (R/R) Follicular Lymphoma (FL) Relapsed or Refractory (R/R) Diffuse Large B-cell Lymphoma (DLBCL) – Phase III	Unknown
Ibritumomab-Tiuxetan (IDEC-2B8) Zevalin	Murine monoclonal anti-CD20 antibody; IgG1 kappa Used in conjunction with radioactive isotope Y-90	Acrotech Biopharma	Approved (USA only)	Non-Hodgkin Lymphoma (NHL) Follicular Lymphoma (FL)	Unknown
Epcoritamab Epkinly (US) Tepkinly (EU)	Humanized monoclonal bispecific anti-CD20/anti-CD3 antibody	AbbVie/Genmab	Approved	Relapsed or Refractory (R/R) Diffuse Large B-cell Lymphoma (DLBCL) Relapsed or Refractory (R/R) Follicular Lymphoma (FL)	Unknown
Glofitamab (RO-7082859) Columvi	Humanized monoclonal bispecific anti-CD20/anti-CD3 antibody (2:1 configuration)	Roche	Approved	Relapsed or Refractory (R/R) Diffuse Large B-cell Lymphoma (DLBCL) Mantle Cell Lymphoma (MCL) – Phase II	Unknown
Imvotamab (IGM-2323)	Recombinant monoclonal bispecific anti-CD20/anti-CD3 antibody; IgM	IGM Biosciences	Phase I/II (terminated)	Relapsed or Refractory (R/R) B-cell Non-Hodgkin Lymphoma (B-cell NHL)	Unknown
Plamotamab (XmAb13676)	Humanized monoclonal bispecific anti-CD20/anti-CD3 antibody; modified Fc domain	Xencor	Phase II (terminated)	Relapsed or Refractory (R/R) Diffuse Large B-cell Lymphoma (DLBCL) – Phase II terminated Chronic Lymphocytic Leukaemia (CLL) – Phase I Rheumatoid Arthritis (RA) – Phase I	Unknown
Divozilimab (BCD-132) Ivlizi	Type I Humanized monoclonal anti-CD20 antibody; IgG1	BIOCAD	Approved (Russia only)	Relapsing Remitting Multiple Sclerosis (RRMS) Systemic Scleroderma – Phase III Neuromyelitis Optica Spectrum Disorders (NMOSD) – Phase III	Unknown
Odronextamab (REGN1979) Ordspono	Human monoclonal bispecific anti-CD20/anti-CD3 antibody; IgG4	Regeneron Pharmaceuticals	Approved (EU only)	Relapsed or Refractory (R/R) Diffuse Large B-cell Lymphoma (DLBCL) Relapsed or Refractory (R/R) Follicular Lymphoma (FL)	Unknown
Ripertamab (SCT400) Anpingxi	Type I Chimeric monoclonal anti-CD20 antibody; IgG1	Sinocelltech	Approved (China only)	Diffuse Large B-cell Lymphoma (DLBCL) Minimal Change Disease (MCD) – Phase III Chronic Inflammatory Demyelinating Polyneuropathy (CIDP) – Phase III	Unknown but likely (similarity with rituximab)

Anti-CD20 drugs target the CD20 surface protein expressed at various stages of B cell development. CD20 is strongly expressed on mature, naïve, and memory B cells and is lost upon B cell differentiation into plasma cells (18).

Although the function of CD20 is not yet fully resolved, it has been proposed to act as a calcium channel and play a role in B cell activation, growth, and differentiation (19). CD20 expression is highly variable across different B-cell malignancies, between patients with the same malignancy, and even among intraclonal subpopulations of individual patients (18). This is in contrast to healthy individuals, where CD20 expression is in a relatively narrow range (20).

Patients treated with anti-CD20 drugs are often candidates for hematopoietic stem cell transplantation (HSCT) due to their underlying hematological diseases or, more recently, are desensitized by anti-CD20 drugs in preparation of a solid organ transplantation. Therefore, samples of these patients are a relevant issue for immunogenetics laboratories.

### 1.3 Anti-CD38: a recent advancement

In recent years, anti-CD38 antibodies such as daratumumab (21) and isatuximab (22) have emerged as significant advancements in the treatment of multiple myeloma (MM), which is a CD20 negative malignancy, and amyloid light chain (AL) amyloidosis.

CD38 (ADP-ribosyl cyclase/cyclic ADP-ribose hydrolase 1) is a transmembrane glycoprotein expressed in different tissues under healthy conditions (23), with elevated numbers in hematological malignancies (21). In contrast to CD20, CD38 is present in both lymphoid and myeloid lineages. Targeting it has shown to be effective in reducing tumor burden. To do so, anti-CD38 antibodies work through multiple mechanisms, including ADCC (21), CDC (21), antibody-dependent cellular phagocytosis (ADCP) (22), immune-mediated cell death, and modulation of the tumor microenvironment (21).

Patients receiving daratumumab or isatuximab often require blood transfusions (24) due to the underlying diseases such as MM. In addition, anti-CD38 antibodies are becoming increasingly popular in the treatment of antibody-mediated rejection (AMR) in organ transplantation (25–28). Further indications are being tested in about 50 clinical trials. Consequently, samples containing anti-CD38 antibodies frequently end up in immunohematology and immunogenetics laboratories.

New antibodies, antibody formats, and antibody-drug conjugates (ADCs) are currently being developed or undergoing clinical trials (**Table 2**). For example, an anti-CD38 antibody has been conjugated with the tubulin-inhibiting drug duostatin 5.2, which induces apoptosis in proliferating tumor cells (29). Another interesting candidate is FTL004, an anti-CD38 antibody with negligible binding to RBCs (30). However, the mechanism behind this discrimination remains unknown, since no difference has been described between RBC CD38 and tumor cell CD38 until now.

**Table 2:** Anti-CD38 drugs approved and in clinical development.

Anti-CD38 drug	Description	Manufacturer	Most advanced clinical status	Indication	Interference
Daratumumab (HLX15) Darzalex	Human monoclonal anti-CD38 antibody, IgG1κ	Janssen	Approved	Multiple Myeloma (MM) Light chain (AL) amyloidosis Neuromyelitis Optica Spectrum Disorders (NMOSD) – Phase II/III Allosensitization, Antibody-mediated Rejection (AMR) – Phase II	IAT FCXM
Isatuximab (SAR-650984) Sarclisa	Mouse chimeric, humanized monoclonal anti-CD38 antibody, IgG1κ	Sanofi	Approved	Multiple Myeloma (MM) T-cell Acute Lymphoblastic Leukemia (T-ALL) – Phase II Light chain (AL) amyloidosis – Phase II	IAT
Felzartamab (MOR202)	Human monoclonal anti-CD38 antibody, IgG1κ	TJ Biopharma /Biogen	Phase III	IgA nephropathy (IgAN) Antibody-mediated rejection (AMR) Multiple Myeloma (MM) – Phase II And others	IAT
Mezagitamab (TAK079)	Human monoclonal anti-CD38 antibody, IgG1κ	Takeda Pharmaceuticals	Phase III	Immune Thrombocytopenic Purpura (ITP) Primary IgA Nephropathy (IgAN) Multiple Myeloma (MM) – Phase II And others	IAT
Erzotabart (Gen3014)	Human monoclonal anti-CD38 antibody, IgG1κ, hexamerization enhanced	Genmab	Discontinued	Multiple Myeloma, Diffuse Large B Cell Lymphoma (DLBCL) Acute Myeloid Leukemia (AML)	IAT
Lumrotatug (CM313)	Humanized mouse monoclonal anti-CD38 antibody, IgG1κ	KeyMed Biosciences	Phase II	Primary IgA Nephropathy (IgAN) Systemic Lupus Erythematosus (SLE) Immune Thrombocytopenia (ITP) And others	IAT
SAR442085	Humanized monoclonal anti-CD38 antibody, IgG1	Sanofi	Phase I	Relapsed/Refractory Multiple Myeloma (RRMM)	Unknown
CID-103	Human monoclonal anti-CD38 antibody, IgG1	CASI Pharmaceuticals	Phase I/II	Chronic Immune Thrombocytopenia (ITP) Multiple Myeloma (MM)	Unknown
FTL-004	Humanized monoclonal anti-CD38 antibody, IgG1κ	Sound Biopharmaceuticals	Preclinical	N/A	Unknown
GBR-1342 (ISB 1342)	Bispecific antibody, BEAT, anti-CD38/anti-CD3	Glenmark Pharmaceuticals	Phase I	Relapsed/Refractory Multiple Myeloma (RRMM)	Unknown
ISB-1442	Bispecific biparatopic antibody format, binding 2x CD38 and 1x CD47	Ichnos Sciences	Discontinued	Relapsed/Refractory Multiple Myeloma (RRMM)	Unknown
ISB-2001	Trispecific antibody, IgG1/3, with anti-CD38/anti-CD3/anti-BCMA	Ichnos Sciences	Phase I	Relapsed/Refractory Multiple Myeloma (RRMM)	Unknown
SG301	Humanized monoclonal anti-CD38 antibody, IgG1κ	Hangzhou Sumgen Biotech	Phase III	Relapsed/Refractory Multiple Myeloma (RRMM) Nephrotic Syndrome in Children – Phase II Systemic Lupus Erythematosus (SLE) – Phase II	Unknown
STI-6129	Antibody drug conjugate: Human monoclonal anti-CD38 antibody + duostatin-5.2	Sorrento Therapeutics	Phase I/II	Relapsed/Refractory Multiple Myeloma (RRMM) Light Chain (AL) Amyloidosis	Unknown
TAK-169	Anti-CD38 engineered toxin body (Shiga-like toxin-A subunit), scFv	Takeda	preclinical	N/A	Unknown
TNB-738	Biparatopic antibody like format with silenced IgG4	Ancora Biotech	Phase I	N/A	Unknown
Y-150	Bispecific antibody, anti-CD38/anti-CD3	Wuhan YZY Biopharma Co., Ltd.	Phase I	Relapsed/Refractory Multiple Myeloma (RRMM)	Unknown

### 1.4 Anti-CD47: the new frontier

Anti-CD47 antibodies represent a newer class of therapeutic agents targeting the “don’t eat me” signal used by cancer cells to evade phagocytosis by macrophages. CD47 is overexpressed in various hematological and solid tumors, making it a promising target for cancer immunotherapy. The blockade of the CD47-signal regulatory protein α (SIRPα) axis enhances macrophage-mediated phagocytosis of tumor cells and has shown potential in preclinical and early clinical trials (31). However, the first-in-class anti-CD47 antibody magrolimab was discontinued in 2024 following the termination of a clinical phase III trial, in which it failed to demonstrate improved survival rates in patients with acute myeloid leukemia (AML) (32). Since then, debates are ongoing whether CD47 remains a viable target for cancer therapy (32).

Still, the clinical development of anti-CD47 drugs moves on (see **Table 3**). The primary indications are myelodysplastic syndrome (MDS), AML, and solid tumors. In addition to classical antibodies, many other anti-CD47 drugs are in development, e.g. SIRPα–Fc fusion proteins and bispecific drugs that additionally target programmed death-ligand 1 (PD-L1), CD19, or CD20 (33). By varying the design of the anti-CD47 drugs, the clinical effect can be tailored to a certain degree, for example aiming at ADCC or phagocytosis (34).

**Table 3:** Anti-CD47 drugs in clinical development.

Anti-CD47 drug	Description	Manufacturer	Most advanced clinical status	Indication	Interference
Magrolimab (Hu5F9-G4)	Humanized monoclonal anti-CD47 antibody; IgG4	Gilead	Discontinued	Acute Myeloid Leukemia (AML) Myelodysplastic Syndrome (MDS)	IAT Reverse Typing
Evorpacept (ALX148)	Fusion protein consisting of SIRPα linked to an IgG1 Fc region	ALX Oncology	Phase II/III	Gastric cancer/Adenocarcinoma	IAT
Lemzoparlimab (TJC4)	Humanized monoclonal anti-CD47 antibody; IgG4	TJ Biopharma Co.	Discontinued	N/A	IAT Reverse Typing
Ligufalimab (AK117)	Humanized monoclonal anti-CD47 antibody; IgG4	Akeso Biopharma	Phase III	Head and Neck Squamous Cell Carcinoma (HNSCC) Acute Myeloid Leukemia (AML) – Phase I/II Higher Risk Myelodysplastic Syndrome (HR-MDS) – Phase II	IAT Reverse Typing
AO-176	Humanized monoclonal anti-CD47 antibody; IgG2	Arch Oncology	Phase I/II	Solid tumor Relapsed/Refractory Multiple Myeloma (RRMM)	Unknown
CPO107/JMT601	Bispecific fusion protein, consisting of SIRPα and anti-CD20 (ofatumumab)	Conjupro Biotherapeutics	Phase II	Primary Membranous Nephropathy B-cell Non-Hodgkin Lymphoma (B-cell NHL) – Phase I	Unknown
HX009	Bispecific fusion protein/antibody, consisting of SIRPα domains and a PD-1 humanized IgG4 antibody	Hanx Biopharmaceutical	Phase II	Advanced Solid Tumor Relapsed/Refractory Lymphoma – Phase I/II	Unknown
IBC0966	Bispecific antibody, anti-CD47 and anti-PD-L1; IgG1	Beijing Sunho Pharmaceutical	Phase I/II	Advanced Malignant Tumors	Unknown
Ontorpacept (TTI-621/PF-07901800)	Fusion protein consisting of SIRPα linked to an IgG1 Fc region	Trillium Therapeutics Pfizer	Discontinued	Relapsed Diffuse Large B-Cell Lymphoma	IAT
Maplirpacept (TTI-622/PF 07901801)	Fusion protein consisting of SIRPα linked to an IgG4 Fc region	Trillium Therapeutics Pfizer	Phase I/II	Diffuse Large B-Cell Lymphoma (DLBCL) Multiple Myeloma (MM)	Unknown
Letaplimab (IBI188)	Fully human monoclonal anti-CD47 antibody; IgG4	Innovent Biologics	Phase I	Advanced Malignancies	IAT Reverse Typing

Anti-CD47 drugs induce transient hemolytic anemia to varying degrees (35, 36). Patients often require transfusions early during therapy, and hence samples containing anti-CD47 drugs will become a relevant issue for immunohematology laboratories. In addition, these patients are often candidates for HSCT, which increases the likelihood that they undergo histocompatibility testing in immunogenetics laboratories.

More recently, anti-CD47 drugs have been developed with the aim of decreasing the binding to RBCs, thereby reducing hematological side effects. For instance, the anti-CD47 antibody ligufalimab has been reported to have a lower incidence of anemia and hemagglutination than magrolimab, which is explained by a different binding angle of the antibody to CD47 (37). Lemzoparlimab is believed to bind to RBC CD47 with lower affinity due to steric hindrance caused by the glycosylation of amino acid residue 32 on mature CD47, a modification that is preferentially found on RBCs (38).

## 2 Affected tests in transfusion and transplantation medicine

### 2.1 Saline agglutination test and serological typing

Serological testing for ABO, RhD, RhCcEe, and K on patient red blood cells (RBCs) is the routine method for blood group typing, which involves hemagglutination reactions between RBC antigens and specific antibodies. This method is fundamental for ensuring compatibility in blood transfusions. Mostly, serological typing is performed in saline agglutination test (SAT) using monoclonal IgM antibodies that directly agglutinate patient RBCs (“forward typing”). This type of typing should not normally be disturbed by therapeutic antibodies which are predominantly in the patient’s serum. However, some therapeutic anti-CD47 antibodies of the IgG type are known to affect reverse ABO typing in SAT (39), where the patient’s plasma is used for direct agglutination of A, B, and O RBCs.

In general, biologics interfere with the SAT by binding to their target antigens on the surface of RBCs and directly inducing agglutination by cross-linking the cells.

### 2.2 Indirect antiglobulin test

The Indirect Antiglobulin Test (IAT) is an essential assay in blood compatibility testing. It is primarily used to detect irregular antibodies in a patient’s serum that could react with donor RBCs. In contrast to the direct agglutination caused by IgM antibodies in the SAT, the IAT uses anti-human globulin (AHG) to induce agglutination of RBCs coated with IgG antibodies.

Both, IAT and the SAT can be performed in tubes, gel or glass beads cards, or microtiter plates. Most of the literature on antibody interference has used card agglutination tests. While the above mentioned techniques differ in the degree to which they are affected by TBI, the principle mechanisms and mitigation strategies are usually transferable.

An additional technical variety of the IAT is the solid phase assay, where RBC ghosts are immobilized on a surface and blood group antibodies are detected after they bound to the immobilized RBCs. In Werfen’s Capture Assay, a commercially available solid phase assay, bound antibodies are detected by indicator RBCs coated with anti-human IgG antibodies.

Biologics interfere with the IAT by binding to their target antigens that are presented on the surface of RBCs. Binding of AHG to the Fc domain of the biologic then induces agglutination. Alternatively, or in addition, biologics that also interfere with the SAT may agglutinate RBCs directly.

### 2.3 Complement dependent cytotoxicity crossmatch

The Complement Dependent Cytotoxicity Crossmatch (CDCXM) is a clinical test used to detect the presence of preformed donor-specific antibodies in the context of organ transplantation. It allows to validate the compatibility between donor and recipient and to assess the risk for a hyperacute allograft rejection following transplantation.

Isolated donor T- and B cells are treated with recipient serum and incubated with complement. Potential donor-specific anti-human leukocyte antigen (HLA) antibodies in recipient serum bind to donor lymphocytes, thereby recruiting complement and leading to cells lysis. A positive crossmatch result indicates an incompatibility and is generally considered a contraindication for organ transplantation (40, 41).

Although this assay is a standard procedure in many clinical laboratories, it has limitations regarding sensitivity compared to newer techniques. As a consequence, the AHG enhanced CDC (AHG-CDC) was developed, increasing assay sensitivity due to an increased rate of crosslinking (42).

Certain therapeutic antibodies and other biologics are able to induce CDC and therefore interfere with CDCXM. In addition, biologics containing an Fc domain may lead to false-positive results in the AHG-CDC despite lacking intrinsic complement-activating capacity.

### 2.4 Flow cytometry crossmatch

Flow Cytometry Crossmatch (FCXM) detects antibodies that bind to donor lymphocytes by utilizing fluorescently labeled secondary antibodies. Compared to CDCXM, FCXM demonstrates higher sensitivity and is particularly useful in detecting low-level antibodies that might not be identified by other conventional methods, thus providing a more comprehensive assessment of compatibility in transplantation settings. Although an improved sensitivity can be advantageous, FCXM is more prone to produce false-positive results and therefore does not always lead to the detection of clinically relevant antibodies (41).

Moreover, some biologics have been reported to interfere with FCXM through binding to target proteins on the cell surface and subsequent detection by secondary antibodies used in this assay, falsely detecting them as anti-HLA antibodies. In contrast to CDCXM, FCXM can also produce false-positive results with therapeutic antibodies or biologics that are less likely to induce CDC (e.g. anti-CD20 Type II antibodies).

## 3 Patterns of therapeutic biologics interference

### 3.1 General aspects

A typical aspect of TBI is the high titers that are observed. For instance, it has been reported that the anti-CD47 antibody magrolimab can reach titers in the range of 100, 000 in the IAT (43), while daratumumab can have titers up to 1, 000 (44).

TBI can still occur months after the last administration of the respective drug. This has been reported for the anti-CD20 antibody rituximab (seven months) (45), the anti-CD38 antibody daratumumab (six months) (46), and the anti-CD47 antibody magrolimab (four months) (43). Based on the pharmacokinetic properties usually desired for biologics, such as persistent circulating drug concentrations and long serum half-lives, we expect that other drugs will exhibit similar behavior. Kim et al. (2026) found indications that the effectiveness of mitigation strategies for TBI may be negatively affected by reduced drug clearance rates and resulting higher drug concentrations (47).

For an overview of which therapeutic biologic is associated with TBI across the different assays, please refer to **Table 4**. An estimate of the surface number of CD20, CD38, and CD47 molecules on the relevant cell types is given in **Table 5**.

**Table 4:** Overview of interference patterns of the biologics and tests discussed in this review.

Biologic class	IAT (cards/tubes)	Capture assay (solid phase IAT)	SAT	DAT	CDCXM	FCXM
Anti-CD20	No interference	No interference	No interference	No Interference	Interference	Interference
Anti-CD38	Interference	Some/weak	No interference reported	Some	No interference reported	Some
Anti-CD47	Interference	Some	Interference(some biologics affect reverse typing, including ABO typing)	Some (48)	No interference reported	No interference reported but likely

**Table 5:** Expression of CD20, CD38, and CD47 on RBCs, T cells, and B cells.

Biologic class	RBC	B cells	T cells
CD20	None	Expressed on the majority of B cells; lost upon differentiation into plasma cells (18)	Usually not expressed on T cells; low expression on a small subset of CD3^+^ T cells (49)
CD38	~500 molecules, strong variation between indivduals (50), decreasing under anti-CD38 treatment	Expressed throughout development, highest in mature B cells (51)	Expression dynamically regulated, high expression associated with T cell activation (52)
CD47	~30, 000 molecules, depending on RH phenotype (53)	Ubiquitously expressed on all B cells; ~20, 000 molecules (54)	Expressed on the majority of mature T cells (55)

### 3.2 Anti-CD20 interference

Anti-CD20 antibodies can interfere with both CDCXM and FCXM by targeting CD20 on B cells. The presence of anti-CD20 antibodies may lead to false-positive results, potentially affecting the assessment of compatibility in organ transplantation (56). Since the tissue distribution of CD20 is largely restricted to B cells (even though a minor subset of T cells may also be CD20-positive (49)), reports of assay interference are limited to B cell CDC- and FCXM. No reports were found for IAT or SAT.

TBI in CDCXM is primarily dependent on the capacity of anti-CD20 antibodies to induce CDC. Among anti-CD20 drugs, rituximab is the most frequently reported biologic interfering with B cell crossmatch assays. It has been shown to produce false-positive results even at concentrations as low as 1 µg/ml (56–58).

Reports on other anti-CD20 antibodies are scarce, although it is reasonable to assume that all type I antibodies share the potential to cause false-positive results in CDCXM due to their innate capability to efficiently recruit complement to the target cell (13). In contrast, anti-CD20 therapeutics may also interfere with FCXM even in the absence of intrinsic CDC activity, where they may be erroneously detected as anti-HLA antibodies.

### 3.3 Anti-CD38 interference

Anti-CD38 antibodies are known to interfere significantly with pre-transfusion tests, particularly the IAT. This interference occurs because CD38 is expressed on RBCs, and the presence of anti-CD38 antibodies can lead to false-positive results in these tests (46). The degree of interference with IAT varies among anti-CD38 antibodies; for example, isatuximab (59) and mezagitamab (60) have been reported to interfere less strongly than daratumumab. The reason for this variability is unclear. No interference has been reported in serological typing or SAT so far (61). In addition, anti-CD38 antibodies can interfere with direct antiglobulin tests (DAT), which involves testing a patient’s RBCs for antibodies already bound to them (62).

Anti-CD38 antibodies can interfere with FCXM (63, 64). No reports were found on interference with the CDCXM. Data on this type of interference is scarce, most likely because therapies involving anti-CD38 antibodies have only recently been introduced in the field of transplantation medicine.

### 3.4 Anti-CD47 interference

Anti-CD47 antibodies present a unique challenge in serological and pre-transfusion testing. CD47 is expressed on all cells, including RBCs, platelets, B cells, and T cells. Anti-CD47 antibodies can interfere with all RBC-based and serological tests, including reverse typing (39). The interference of anti-CD47 drugs in the IAT is characterized by strong reactions with very high titers. Anti-CD47 drugs with reduced binding to RBCs that have been developed to reduce hematological side effects also show TBI in immunohematology (43).

We have found no reports that investigated the effect of anti-CD47 drugs on FCXM; however, it is highly likely that they interfere with this assay. Interestingly, anti-CD47 drugs may not interfere with CDCXM, although CD47 can be found on B cells and T cells. CDC has not been reported for anti-CD47 antibodies so far (65).

## 4 Remedies and solutions

From a technical point of view, the main difficulty in dealing with TBI is the high concentration of these drugs in the serum, which can easily reach 1 mg/ml or more. Given an average IgG concentration of approximately 10 mg/ml in human serum (66), about 10% of the circulating antibodies are therapeutic biologicals in these cases. This poses a significant challenge for technical solutions to TBI, as the methods employed must be highly effective in order to completely block the interference.

The main organizational challenge for laboratories may be obtaining information about the patient’s therapeutic regimen. This includes current medication, as well as medication taken within the last six or even twelve months. False-positive reactions caused by therapeutic biologics that are not disclosed or known may only cost time in the best case, but in the worst case they may impact patient safety.

### 4.1 Matching

Matching in immunohematology ensures the donor´s red cells are negative for clinically most relevant antigens the patient does not carry on his erythrocytes. For patients under anti-CD38 therapy receiving blood transfusions, an extended antigen matching is often performed, covering most or all of RhD, RhCcEe, K, Fy(a/b), Jk(a/b), S, and s antigens (67, 68). This strategy avoids hemolytic transfusion reactions caused by undetected antibodies and prevents alloimmunization. The antigen typing of the patient can be performed before starting the therapy or by genotyping at any stage of the therapy. In transplantation, matching of HLA is the key factor in preventing transplant rejection.

Matching is limited by the availability of blood or transplants and the depth of patient and donor typing, which set the boundaries for the practical impact for this strategy. In this context, red cell genotyping has emerged as an important complementary—and sometimes alternative—approach to conventional serological testing. However, genotyping also has its limitations, including cost, turnaround time, and occasional discrepancies between genotype and antigen expression, meaning that serological and molecular approaches are often most effective when used together (69). The situation is less complex for HLA typing, where genotyping is already state of the art.

### 4.2 Antigen negative cells

CD38 expression on RBCs is considerably variable between individuals (50) but is independent from RBC age (46). Neonatal RBCs may be deficient of CD38 and have been used in the IAT to avoid the interference by daratumumab (70). RBCs with the rare In(Lu) phenotype do not react with daratumumab (71). In addition, it is known that RBCs of patients under daratumumab treatment have low CD38 surface expression (72). All of these sources can be used to formulate CD38 negative RBC panels for antibody screening and identification (73). However, there are important practical limitations to consider: Neonatal RBCs and In(Lu) RBCs have altered expression of blood group antigens compared to standard panel cells, due to developmental immaturity or the In(Lu) phenotype itself, respectively, and require stringent typing. In addition, all of these RBCs are not easy to obtain for routine laboratories (71–74).

Antigen negative cells are only applicable to CD38 and the IAT. To the authors, no similar approaches are known for CD20 and CD47, and no such approaches are known for CDCXM or FCXM. Mutations of the Rh-associated glycoprotein (RhAG) can lead to a strong reduction of CD47 expression on RBCs (75), however, we have found no reports that these cells have been used for IAT.

### 4.3 Antigen denaturation by disulfide bridge reduction

Dithiothreitol (DTT) treatment has emerged early as a method to overcome the interference caused by anti-CD38 antibodies in pre-transfusion testing (62). DTT is a reducing agent that denatures CD38 on the surface of RBCs by cleaving disulfide bonds. It has been shown that this treatment destroys the epitopes of daratumumab and isatuximab on CD38, thus preventing their binding and eliminating pan-reactivity in the IAT (59).

The standard procedure for DTT treatment of RBCs, as reported by Chapuy et al. (2016) and subsequently recommended in several transfusion society guidelines, uses 200 mM DTT for CD38 denaturation (76). There are conflicting reports regarding the effectiveness and reproducibility of this method. While Chapuy et al. used two samples spiked with daratumumab at a concentration of 10 µg/ml and reported a 100% success rate among 25 study centers (76), Habicht et al. (2023) achieved a success rate of only 68.2% for DTT treatment of 50 patient samples containing daratumumab (77). Daratumumab can be present in patients’ serum at concentrations of 1 mg/ml or higher.

In addition, DTT treatment is unspecific and can destroy or weaken clinically relevant antigens from the KEL (78), DO (79), VEL (80), and LU blood group systems, and the less relevant antigens in the YT (78), JMH, LW, IN, CR, RAPH, and KN blood group systems.

Recent studies have shown that lower concentrations of DTT can be effective in eliminating anti-CD38 antibody interference while preserving the integrity of other blood group antigens (78). For instance, a variant of the DTT treatment has been described with the “Osaka method”, where 10 mM DTT at pH 7.3 were sufficient for CD38 denaturation (81). Izzaguirre et al. (2020) needed 40 mM DTT at the suboptimal pH 7.0 without washing steps (82), Furumaki et al. (2019) were able to show CD38 denaturation with as little as 7.5 mM DTT at pH 8.0 (83). These advanced protocols may make DTT treatment a more viable option, depending on their effectiveness and reproducibility, and their effect on other blood group antigens.

Zhou et al. (2021) have used 2-Mercaptoethanol (2-ME) as a reducing agent and alternative to DTT in pre-transfusion testing. In their study, 2-ME showed effects and limitations comparable to DTT (84).

In any case, it remains to be seen if the interference of all anti-CD38 drugs can be remedied by treatment with reducing agents. Antibodies that bind a linear, non-conformation dependent epitope on CD38 may interfere with pre-transfusion testing even after the disruption of the tertiary structure of CD38 by DTT or 2-ME. A study with human and macaque CD38 showed that some anti-CD38 antibodies react to DTT-denatured CD38 (85), and recent findings suggest that the epitope of felzartamab is partially DTT-resistant (86).

DTT does not mitigate the interference caused by anti-CD47 antibodies or anti-CD20 antibodies but is commonly used in CDCXM and FCXM to distinguish DTT-sensitive IgM from DTT-resistant IgG antibodies.

### 4.4 Proteolytic digestion of antigens

Proteolytic digestion of RBCs with trypsin or papain is – like DTT treatment – a common practice in immunohematology laboratories. Like DTT, this treatment is rather unspecific and leads to the destruction of a variety of blood group antigens. For trypsin, it has been reported that antigens in the CH/RG, XG, IN, JMH, MNS, GE, LU, KN, and DO blood group systems are affected (87); papain has a very similar pattern but notably also affects FY antigens. On the other hand, some antibodies react stronger after protease treatment because the enzymes cleave sialic acid-rich glycoproteins from the RBC surface, reducing the negative surface charge and allowing cells to come closer together.

To our experience, reactions with the anti-CD38 antibodies daratumumab and isatuximab in the IAT are enhanced after papain treatment. Thus papain treatment is not useful for the mitigation of their interference in the IAT. However, anti-CD38 does not react in the SAT with untreated and papain treated test cells. Thus the SAT may be useful to detect underlying IgM antibodies detectable by direct agglutination when using papain treated red cells (87–89).

Chapuy et al. were able to mitigate daratumumab induced interference by using trypsinated RBCs (62) and Ibeh et al. reported successful inhibition of daratumumab interference with trypsinated RBCs in 98.4% of all cases across two laboratories in tube testing (82).

The advantage of this approach is that enzyme-treated cells are easy to generate and, in case of papain, are even commercially available; the broad and unspecific destruction of blood group antigens is a major drawback that requires careful evaluation of the results obtained.

CD47 seems not to be sensitive to treatment with trypsin, papain, or α-chymotrypsin (39).

Pronase, a mixture of proteases isolated from the extracellular fluid of Streptomyces griseus, is used to improve the specificity and sensitivity of FCXM by reducing nonspecific antibody binding and revealing hidden epitopes. In B cell FCXM, pronase treatment (typically 0.5–1 mg/ml) prevents false-positive results by removing Fc receptors and surface immunoglobulins (90, 91). Higher concentrations of 2–3 mg/ml have been used to mitigate rituximab interference by removing CD20 from the cell surface (91, 92). However, the technique has notable limitations: it can reduce HLA expression on B and T cells significantly, potentially leading to false-negative results in FCXM (92–94). In addition, a study cautions about potential interference, especially in HIV-positive patients (95). Careful optimization of pronase concentration is crucial to balance sensitivity and specificity.

### 4.5 Adsorption

Allogeneic adsorption (alloadsorption) involves incubating patient plasma with RBCs or other cells of a known phenotype. Antibodies that bind to the cells used for adsorption can thus be depleted from the sample, allowing for detection of underlying antibodies.

So far, adsorption approaches in pre-transfusion testing using RBCs have not been very successful in mitigating TBI (62). Therefore, alternative sources for antigens have been explored. For example, the B cell line Daudi has been used as a source for CD38 containing membranes (96) and for the adsorption of anti-CD47 drugs (97). A more directed adsorption method has been applied by Ehrend et al. in 2021. By generating recombinant cells that present CD38 on the cell surface, they were able to reduce the daratumumab concentration to a point where the reaction in the IAT became negative. However, with higher titers they required more than one adsorption cycle (98).

Anti-CD47 drugs may require even more elaborate adsorption strategies. For instance, Velliquette et al. (2019) tried to adsorb the anti-CD47 antibody magrolimab on RBCs or platelets and needed at least four adsorption cycles (39). In contrast, Kim et al. (2020) tried to adsorb magrolimab in the presence of PEG and failed even after six rounds of adsorption (99). This is in line with the observation that anti-CD47 drug interference is usually stronger than anti-CD38 in pre-transfusion testing.

No reports on adsorption for CDCXM or FCXM were found.

### 4.6 Soluble antigens

The use of soluble antigens has shown promise in mitigating TBI caused by anti-CD38 (46, 100) and anti-CD47 (99) antibodies. This approach involves adding a soluble form of the target antigen to which the therapeutic antibodies can bind directly within the patient sample. This prevents the antibodies from interacting with cell surface antigens during testing. For anti-CD38 antibodies such as daratumumab, soluble CD38 can effectively reduce interference in pre-transfusion tests. However, this method has its limitations, particularly with regard to interference with anti-Fy antibodies (100). Research is ongoing to explore the potential of soluble antigens in managing interference caused by anti-CD47 antibodies as well (99).

A further limitation of this approach is that therapeutic antibodies usually reach very high serum concentrations of 1 mg/ml or even higher. The soluble antigens added have to reach a concentration at least in the same order of magnitude to bind all antibodies in solution effectively. Depending on the system, this ratio may become even significantly higher. For instance, Kim et al. (2020) needed 50- to 100-fold molar excess of soluble CD47 to mitigate the interference of evorpacept (99), which may quickly reach limits of protein solubility.

A new approach, consisting of a mixture of adsorption and soluble antigens, has used magnetic beads coated with cell membranes or recombinant CD47 to deplete magrolimab at concentrations below 1 µg/ml (101).

### 4.7 Anti-idiotype antibodies

Anti-idiotype antibodies bind to the paratope of a target antibody, thereby preventing it from binding to its original epitope. These antibodies are often developed alongside therapeutic antibodies in order to monitor pharmacokinetics and anti-drug antibodies.

Examples of anti-idiotype antibodies include those for the anti-CD38 antibody daratumumab (102, 103), for the anti-CD47 drug evorpacept (104), and for the anti-CD20 antibody rituximab (105, 106). Like soluble antigens, they are added to the specimen before testing. They have been successfully employed to mitigate interference in IAT (102, 103), FCXM (106, 107), and CDCXM (105).

Anti-idiotype antibodies are usually highly specific, meaning they can only be used to neutralize their respective target. For instance, an anti-daratumumab antibody typically does not inhibit other anti-CD38 antibodies. This specificity poses practical challenges in clinical laboratories, as precise knowledge of the patient’s medication history is required and anti-idiotype antibodies may not be available for a particular therapeutic antibody.

Surprisingly, a report indicates that some blood group antibodies, specifically anti-Le and anti-M antibodies, where not detected any more when using anti-idiotype antibodies (108). This problem was confirmed by a study where 3.6% of all irregular antibodies were not detected anymore after the use of an anti-daratumumab antibody (102).

### 4.8 IgG subtype specific AHG or secondary antibodies

Since current therapeutic antibodies are monoclonal and of a specific IgG subtype, strategies to mitigate TBI in pre-transfusion testing have included the use of AHG that selectively recognizes certain IgG subtypes. Specifically, IgG4-,,blind” AHG has been used to overcome magrolimab interference, an antibody of the IgG4 subtype. The AHG coupled to the indicator RBCs in Werfen’s Capture Assay is also claimed to be IgG4-blind. While reducing interference significantly, both approaches do not lead to completely negative results for magrolimab and other anti-CD47 antibodies (43), probably due to the high antigen density of CD47 on RBCs.

In principle, this approach is transferable to FCXM. Secondary antibodies that are specific for certain IgG subtypes or that have been negatively adsorbed for one subtype could avoid background from specific therapeutic antibodies. This solution is not applicable for CDCXM, though. Complement activation is dependent on IgG subtypes, primarily IgG1 and IgG3, and less IgG4 (109). It does not appear possible to modify complement in a way that this is altered. In fact, therapeutic antibodies are often designed to induce CDC.

### 4.9 Polybrene

Strictly speaking, the manual polybrene test is not an IAT and therefore not in the scope of this review. Polybrene is a cationic polymer. In the manual polybrene test (MPT), it is used to neutralize the natural negative surface charge of RBCs. This reduction in repulsion allows RBCs to come into close proximity, facilitating the cross-linking of cells by antibodies (if present) and resulting in visible agglutination. After addition of a neutralizing reagent like sodium citrate, RBC aggregates are resuspended and only antibody-mediated agglutinates remain.

The MPT has varying sensitivity to irregular antibody specificities compared to the IAT. Several studies have shown that the MPT is far less sensitive to daratumumab and isatuximab than the IAT (84, 110, 111). However, the MPT is known to be insensitive to KEL blood group system antibodies.

This approach cannot be used for CDCXM or FCXM.

### 4.10 AHG masking

A mitigation strategy for the IAT is the blockage of CD38 on the surface of RBCs with anti-CD38 antibodies that are afterwards blocked with AHG. This approach was described initially by Chinoca Ziza et al. in 2019 (112). However, the method has been received as cumbersome. Nedumcheril et al. mentioned that it “has limitations which include technical difficulty, unavailability of anti-CD38, and need to titrate anti-CD38 prior to testing” (113). We have found no reports that this approach has been tested for anti-CD47 drugs.

### 4.11 Antigen masking

Antigen masking techniques use specific agents to block the binding sites of therapeutic antibodies on RBCs or other cells used in diagnostic tests. The masking molecule does not carry a human Fc domain and therefore cannot interfere with the tests. This approach has shown effectiveness for various antibodies, including anti-CD38 (50, 77, 114), anti-CD20 (115), and anti-CD47 (43, 99). So far, mainly Fab or F(ab’)2 antibody fragments have been used, however in the case of CD47, its natural ligand SIRPα has also been utilized (43). A more recent approach uses a recombinant rabbit monoclonal anti-CD38 antibody for serological testing (116).

A commercially available agent for the antigen masking IAT (AMIAT) has been introduced into routine pre-transfusion testing by several labs. Tenorio et al. (2019) and Constantin et al. (2019) described it as easy and quick (117, 118). Kim et al. (2026) were able to inhibit 100% of 22 samples with daratumumab with it, compared to only 19 negative results with DTT-treated cells (47). These findings are in line with other reports that show superiority over DTT (77, 119).

For FCXM or CDCXM, antigen masking has not yet been implemented into routine testing, but a similar approach has been used by Baig et al. (2020) for the flow cytometric granulocyte immunofluorescence test (Flow-GIFT). They were able to mitigate daratumumab interference with Flow-GIFT by masking CD38 on granulocytes with the mouse anti-human-CD38 antibody HB7 (120).

The versatility of antigen masking makes it a promising solution for managing interference caused by a wide range of biologicals.

## 5 TBI in the future

### 5.1 Combination therapies, bi- and multispecific biologics

Combining antibodies against CD20, CD38, and/or CD47 offers promising new options for treating severe diseases. For instance, a recent case report has described the use of anti-CD20 and anti-CD38 antibodies for the treatment of lupus nephritis patients (121). Combination therapies involving anti-CD38 antibodies and anti-CD47 drugs for the treatment of relapsed and/or refractory multiple myeloma (RRMM) are either thought of (felzartamab and lemzoparlimab) (122) or already studied clinically (isatuximab and evorpacept, NCT04643002 (clinicaltrials.gov)). The anti-CD47 antibody CC-90002 has been used in combination with rituximab to treat relapsed and/or refractory NHL (123).

Bi- and multispecific drugs, which are currently in development, take this strategy a step further. The bispecific antibody IMM-5605, targeting both CD38 and CD47, has shown promise in the treatment of cancer in mouse models (124). ISB-1442, a bispecific and biparatopic anti-CD38 and anti-CD47 molecule, was intended for the treatment of MM (125) but has been discontinued.

Several CD20xCD47 bispecific antibodies or fusion proteins are either in development or already in clinical studies. These include two SIRPα anti-CD20 fusion proteins, CPO-107 (126) and IMM-0306 (127), and bispecific antibodies such as BMS-986358 (128) and RTX-CD47 (129). HuNb1-Rituximab is a fusion protein comprising an anti-CD47 nanobody and rituximab (130). All of these are intended for treating B cell malignancies, such as NHL or CLL.

IMM-0306 has been designed to bind to RBCs with low affinity only (127). However, it is unclear how this affects interference in serological testing.

### 5.2 Multivalent antibodies

IgM antibodies are the classical multivalent antibodies that lead to direct agglutination in pre-transfusion tests and that can trigger strong CDC. Pre-clinical and clinical research on IgM antibodies as therapeutic agents has so far mostly focused on targets such as glycans, glycolipids, or lipopolysaccharides that profit from the higher avidity of IgM compared to IgG (131), and for which TBI has not yet been reported.

Nevertheless, multivalent antibodies may still become an issue for diagnostics in transfusion and transplantation medicine. Genmab is developing hexamerization-enhanced antibodies, such as the anti-CD38 antibody erzotabart (GEN-3014) (132). The IgG1 Fc of this molecule has been modified so that Fc-Fc interactions increase upon antigen binding (133). This enables an IgG1 hexamer to be formed, which triggers direct cell killing via ADCC, ADCP, and CDC.

Multivalent antibodies pose a serious potential complication for the SAT and the IAT, because they may directly agglutinate RBCs. The intended increase in cell cytotoxicity may directly impact TBI in the CDCXM.

### 5.3 Imlifidase

A different form of TBI may be induced by imlifidase, which was approved by the EMA in 2020. Imlifidase is a therapeutic protease derived from *Streptococcus pyogenes* which specifically cleaves the Fc domain from IgG antibodies. It is used to treat highly sensitized patients in order to facilitate HLA-incompatible kidney transplantations (134). Osmani et al. (2025) reported that treatment with imlifidase in one patient resulted in negative IAT results. A previously confirmed anti-K antibody could no longer be detected directly after treatment and three months afterwards (135). While it is worth discussing whether this finding is a false negative or rather a true negative result, and thus whether this is interference after all, its clinical significance is unclear.

Huang et al. have predicted that imlifidase may also interfere with the AHG-CDC (134). Generally, residual single-cleaved IgG after imlifidase treatment can still produce positive signals in Luminex single antigen bead assays, FCXM, and CDCXM. These assays, however, function as practical readouts of whether imlifidase has achieved sufficient IgG cleavage and crossmatch conversion (136).

### 5.4 Other antigen targets: CD52

CD52, also known as CAMPATH-1 antigen, is a small glycoprotein of 12 amino acids that is present on the surface of mature lymphocytes, monocytes, dendritic cells, and others. Alemtuzumab is the only anti-CD52 antibody approved for clinical use, with indications in multiple sclerosis (MS) (137) and previously in CLL (138). RD04-CD52-V01 is a recombinant humanized anti-CD52 monoclonal antibody in phase I trials for a broad range of lymphoid malignancies, including CLL, T cell-prolymphocytic leukemia (T-PLL), and various NHL subtypes (ClinicalTrials.gov ID NCT05557903).

Alemtuzumab has been reported to interfere with FCXM (107, 139). However, it has only attracted marginal attention regarding TBI so far, possibly because fewer patients have been treated with this drug than, for example, rituximab, due to its narrower indications. With new anti-CD52 antibodies in the clinical pipeline, TBI by anti-CD52 drugs may become more relevant.

### 5.5 Future antigen targets: CD44

CD44 is a cell surface glycoprotein, which is expressed on normal cells, and overexpressed on cancer cells (140, 141). Anti-CD44 antibodies are in preclinical and early clinical development for various conditions, including cancers such as acute myeloid leukemia, head and neck cancers, and ovarian cancer, as well as inflammatory diseases (142–146). Several isoforms, commonly called variants, of CD44 exists, generated by variable splicing of exons 6 to 15 (141). The shortest variant, CD44s, is probably the only form expressed on RBCs. Lymphocytes carry longer CD44 variants depending on the cell type, as well as the physiological and/or pathological condition (147). Some of the anti-CD44 drugs in development specifically target these variants. One example is the anti-CD44v6 antibody bivatuzumab (148).

None of the identified clinical trials have progressed beyond phase I, even though the first trials began in 2002. This suggests that this therapeutic approach may be more challenging than initially anticipated (149). Nevertheless, research is ongoing and new drug candidates are currently being developed. Anti-CD44 drugs have the potential to interfere with all the tests discussed in this review. However, the targeted CD44 variant of the drugs vary, and so may the interference of each drug.

Interestingly, CD44 is also the carrier of the IN blood group system (also known as Indian) (150), which currently has six known antigens (151). The IN antigen is DTT sensitive and soluble recombinant IN-antigens have already been studied for their ability to inhibit anti-IN antibodies (152).

### 5.6 Future antigen targets: CD36

Anti-CD36 antibodies are being investigated as a potential therapeutic option, particularly in the field of oncology (153). Studies show that an antibody against the lipid and protein receptor CD36 can inhibit the metastasis of various tumors in preclinical models (154). However, it will be several years before these approaches can be used clinically in humans, as they are currently still in the preclinical or very early development phase.

CD36 is a surface protein that is found not only on tumor cells, but also on platelets, monocytes, macrophages, and, to a lesser extent, on RBCs. Naturally occurring anti-CD36 antibodies are known to cause immunohematological complications, for example fetal/neonatal alloimmune thrombocytopenia (FNAIT) (155) and platelet transfusion refractoriness (156). CD36 has recently been recognized as a new blood group system (157).

Therefore, it is likely that therapeutic anti-CD36 antibodies may interfere with pre-transfusion diagnostics, particularly the IAT. However, CD36 expression on adult RBCs is low, so interference may be weak or sporadic. Similarly, only low CD36 expression has been reported on peripheral T cells (158) and varying expression on B cells (159), indicating that CDCXM and FCXM may not be affected or may only be affected to a small degree.

### 5.7 Future antigen targets: CD71, CD55, CD59, and CD99

CD71 (transferrin receptor 1) is a target of therapeutic biologics for the treatment of cancer (73). Two molecules are currently being or have been recently investigated in clinical trials: INA03, an IgG4 drug conjugate (160), and CX-2029, a so-called probody that gets cleaved to an active state in the tumor environment (161). As RBCs are CD71 negative, the potential of these drugs to interfere with pre-transfusion testing is rather low. B cells and T cells carry CD71 but both drugs are unlikely to initiate CDC, so they are probably not relevant for CDCXM. INA03 and CX-2029 could affect FCXM, though.

Anti-CD55 antibodies are in preclinical development for the treatment of solid tumors (162, 163), but no current clinical trials were identified. CD55 is known as the carrier molecule of the CROM blood group system (164); thereby demonstrating the potential for interference of anti-CD55 antibodies in pre-transfusion testing. The expression of CD55 on B cells and T cells varies depending on the cell subset and its physiological state (165), indicating that at least some interference with CDCXM and FCXM is to be expected. Recombinant, soluble CD55 has been successfully used to inhibit anti-CROM antibodies in agglutination assays (166).

CD59 and CD99 have been discussed as therapeutic targets that could interfere with pre-transfusion tests (73) but we were not able to identify recent clinical or preclinical developments in this regard.

### 5.8 B cells only: CTLA-4 Igs

CTLA-4-Igs are cytotoxic T-lymphocyte-associated protein 4 immunoglobulin fusion proteins. These interact with B7–1 and B7–2 on antigen presenting cells, such as B cells, and block the CD28/B7 costimulatory pathway (167). CTLA-4-Ig includes two approved drugs, abatacept for rheumatoid arthritis (168) and belatacept for renal transplantation (169), and one candidate in early trials, RG2077 for multiple sclerosis (170). Abatacept and belatacept are Fc IgG1 fusion proteins, RG2077 is a Fc IgG4 fusion protein. Thus, these molecules have the potential to interfere with CDCXM and FCXM with B cells. However, even though abatacept and belatacept have been approved for several years, no reports of interference could be found, indicating that this is only a hypothetical risk.

## 6 Conclusion

The increasing use of therapeutic antibodies and biologics in clinical practice has brought significant advancements in treatment options but also introduced new challenges in serological and pre-transfusion testing. SAT, IAT, FCXM, and CDCXM are all affected to varying degrees by TBI. Understanding the specific interference patterns of anti-CD20, anti-CD38, and anti-CD47 drugs is crucial for developing effective strategies to manage their impact on diagnostic tests. The examples of combination therapies, bispecific antibodies or multivalent antibodies highlight that navigating TBI may become even more complex in the future.

The good news is that effective mitigation methods are available or under development. Additionally, the integration of molecular testing methods, such as genotyping, may offer new avenues for overcoming the limitations of traditional serological methods, particularly in complex cases.

In conclusion, the fields of transfusion and transplantation medicine must continue to adapt and innovate to address the challenges posed by TBI. By doing so, we can ensure the continued safety and efficacy of blood transfusions and organ transplantations in the era of targeted immunotherapies.
